# PROTOCOL: Searching and reporting in Campbell Collaboration systematic reviews: An assessment of current methods

**DOI:** 10.1002/cl2.1208

**Published:** 2021-12-14

**Authors:** Sarah Young, Alison Bethel, Ciara Keenan, Kate Ghezzi‐Kopel, Elizabeth Moreton, David Pickup, Zahra A. Premji, Morwenna Rogers, Bjørn C. A. Viinholt

**Affiliations:** ^1^ University Libraries Carnegie Mellon University Pittsburgh Pennsylvania USA; ^2^ NIHR CLAHRC South West Peninsula (PenCLAHRC) University of Exeter Medical School Exeter UK; ^3^ Campbell UK & Ireland, Centre for Evidence and Social Innovation Queen's University Belfast UK; ^4^ Albert R. Mann Library Cornell University Ithaca New York USA; ^5^ University of North Carolina at Chapel Hill Chapel Hill North Carolina USA; ^6^ Centre for the Study of Learning and Performance Concordia University Montréal Québec Canada; ^7^ Libraries and Cultural Resources University of Calgary Calgary Canada; ^8^ NIHR PenCLAHRC, Institute of Health Research University of Exeter Medical School Exeter UK; ^9^ VIVE—The Danish Center for Social Science Research Copenhagen Denmark

## Abstract

This is the protocol for a Campbell review. The aim of this study is to comprehensively assess the quality and nature of the search methods and reporting across Campbell systematic reviews. The search methods used in systematic reviews provide the foundation for establishing the body of literature from which conclusions are drawn and recommendations made. Searches should be comprehensive and reporting of search methods should be transparent and reproducible. Campbell Collaboration systematic reviews strive to adhere to the best methodological guidance available for this type of searching. The current work aims to provide a comprehensive assessment of the quality of the search methods and reporting in Campbell Collaboration systematic reviews. Our specific objectives include the following: To examine how searches are currently conducted in Campbell systematic reviews. To identify any machine learning or automation methods used, or emerging and less commonly used approaches to web searching. To examine how search strategies, search methods and search reporting adhere to the Methodological Expectations of Campbell Collaboration Intervention Reviews (MECCIR) and PRISMA guidelines. The findings will be used to identify opportunities for advancing current practices in Campbell reviews through updated guidance, peer review processes and author training and support.

## 1 BACKGROUND

### 1.1 Description of the problem or issue

The systematic search for literature is arguably the most important part of a systematic review, as it provides the foundational data set for the review from which the conclusions are derived. Search methods should be as comprehensive as possible including searches of relevant research databases as well as sources of gray literature. Searches should also be well‐documented, transparent and reproducible. Existing systematic review guidance and standards provide steps and reporting checklists that authors can follow to achieve consistent and reproducible searches. For example, a recent update of the Preferred Reporting Items for Systematic Reviews and Meta‐Analyses (PRISMA) provides detailed guidance on the components of a search that should be included with any published systematic review (Page et al., 2021). These include a full search strategy for every database, registry, and website searched; a description and justification of limits applied; and an indication of if and how the search was peer‐reviewed, among several other reporting items related to the search. In addition to these items, an extension to PRISMA, PRISMA‐S, was published specifically for search reporting (Rethlefsen et al., 2021). For Campbell systematic reviews, authors are often directed to the Methodological Expectations of Campbell Collaboration Intervention Reviews (MECCIR) Reporting Standards (Methods Group of the Campbell Collaboration, 2019) and the Campbell Collaboration's guide to information retrieval (Kugley et al., 2017). These standards mandate that all sources searched including database name, platform and date coverage be listed, as well as exact search strategies for each database searched, among other requirements.

Despite this guidance and the existence of the PRISMA checklist and MECCIR standards, search methods in many published systematic reviews are not reproducible (Faggion et al., 2018; Koffel & Rethlefsen, 2016; Toews, 2017). For example, in a study of methods transparency in psychology meta‐analyses, Polanin et al. (2020) found that only half of the 150 studies analyzed reported the dates of searches, and roughly one‐quarter of studies failed to report the terms used for searching. Reproducibility and transparency in systematic reviews is important to demonstrate the comprehensiveness of the search. Reproducible and transparent methods highlight and reduce potential biases across the entire review process, from the search and selection of studies to data extraction, analysis and interpretation. Reproducible and transparent searches also highlight gaps that can be addressed in future work and facilitate the updating of systematic reviews, which is critical to incorporate new evidence as it emerges and to update recommendations and guidance that come from systematic reviews.

Authors of Campbell Systematic Reviews can request editorial and methodological support and guidance to conduct searches. In some cases, information specialists or librarians with training in systematic searching are included as co‐authors or consultants, though such involvement is not currently required. Campbell systematic reviews and protocols also undergo a peer review process that includes information specialists with expertise in systematic review searching. Information specialist peer‐reviewers assess the appropriateness of the searched databases and gray literature, the usage of subject headings, keywords and Boolean operators, and the reporting of search methods, among other factors. The current guidance and documentation used for the peer review of Campbell review searches is in need of an update, due to advancement of methods and technology, and this update should reflect current best practices and guidance for comprehensiveness and reproducibility.

### 1.2 Description of the methods being investigated

The search strategy and retrieval methods in a systematic review encompass a range of activities, largely in the early phases of the review process. Thus, this study will assess the current methods and reporting practices for source selection, searching, and reference management. Another aspect of searching for systematic reviews is search performance, often assessed with measurements of precision and sensitivity. This aspect of searching is outside the scope of the current study.

## 2 WHY IT IS IMPORTANT TO DO THIS REVIEW

No broad systematic assessment of the search methods used in Campbell reviews has been published to date and the extent to which Campbell review searches adhere to current guidelines is unclear. Wang et al. (2021) recently conducted a review of methodological and reporting characteristics of Campbell systematic reviews. They examined reviews published since 2011 and assessed whether the introduction of the MECCIR standards impacted methods and reporting across the systematic review process, including some aspects of searching (reporting of information sources, search dates and search terms). They found evidence of incomplete search reporting including a lack of complete source date coverage reported in just under half of the reviews, hand‐searching methods reported in only 48% of studies, and the search used for conference abstracts reported in only 16% of studies. The current study aims to build on this study focusing on search methods and reporting with an in‐depth examination of all search methodological and reporting aspects necessary to adhere to standards and to make searching reproducible.

Systematic reviews outside of the Campbell Collaboration have been assessed for quality of search methods and reporting. Methodological research on published Cochrane reviews shows deficiencies in adherence to search strategy construction and reporting standards. Franco et al. (2018) took a random sample of 70 Cochrane reviews published after 2015 and evaluated the design and reporting of their search strategies against *Cochrane Handbook* standards. They found problems in the design of 73% of the search strategies that they examined. Similarly, Yoshii et al. (2009) analyzed 65 Cochrane reviews and found that none of them adhered to all seven elements of an electronic database search strategy listed in the *Cochrane Handbook*. The quality of methods in qualitative research syntheses published by the Joanna Briggs Institute was assessed, with over one‐quarter of reviews lacking clear information about when the search was run (Munn et al., 2021).

Moreover, the application of emerging methods, such as machine learning for search strategy development or automation of deduplication steps, has not been assessed and is not currently accounted for in the search peer review process. Thus, this study aims to contribute to an understanding of current practices and assess search methods and reporting quality in Campbell Collaboration systematic reviews. This includes highlighting potential biases to which Campbell Collaboration systematic reviews may be subject as a result of the search methods applied. This study will be used to inform guidance for Campbell review authors and to update the search peer review process and documentation. Through an informed update of guidance and the peer review process, the overall search quality and reproducibility of Campbell systematic reviews can be improved.

## 3 OBJECTIVES

The aim of this study is to comprehensively assess the quality and nature of the search methods and reporting across Campbell systematic reviews. Our specific objectives include the following:

1. To examine how searches are currently conducted in Campbell systematic reviews.
2. To identify any machine learning or automation methods used, or emerging and less commonly used approaches to web searching.
3. To examine how search strategies, search methods and search reporting adhere to the MECCIR and PRISMA guidelines.

## 4 METHODS

### 4.1 Criteria for considering studies for this review

#### 4.1.1 Types of studies

All systematic reviews published since January 2017 will be included. Protocols, methods papers, commentaries, editorials and other types of evidence synthesis (e.g., evidence and gap maps, mega maps) will be excluded. Updates to previous systematic reviews will also be included. If the update indicates a change to the search methods, data will be extracted from the updated systematic review and will be included in subsequent analyses.

We chose January 2017 to reflect our interest in examining current search practices. As search methods have evolved with rapid changes in the information landscape and the application of automation and machine learning to evidence synthesis, reviews published in the last three years should provide an accurate picture of current practices. That being said, the date of publication is not an accurate representation of when a search was conducted. Thus, searches conducted well before 2017 (but published since January 2017) will be included as a point of comparison to more recent methods. Notably, the Campbell Collaboration's current guidance document for information retrieval was published in February 2017 (Kugley et al., 2017). With the publication of this document, practices may have changed to reflect this new guidance. Of the 61 systematic reviews published since January 2017 as of the writing of this protocol, 28 of those reviews indicate searches conducted after the publication of the current guidance. Thus, our sample will provide a good approximation of pre‐ and post‐guidance practices.

### 4.2 Search methods for identification of studies

The search methods for identifying studies is outlined below.

#### 4.2.1 Electronic searches

We will search the Campbell Systematic Reviews journal on the Wiley Online Library website to identify all systematic reviews published since January 2017. To do this, we will hand search the tables of contents of all Campbell Systematic Reviews issues from 2017 forward.

### 4.3 Data collection and analysis

#### 4.3.1 Selection of studies

Given the straightforward nature of the selection criteria (i.e., systematic reviews or updates to systematic reviews published in January 2017 or later), a single reviewer only will carry out the electronic search and selection of studies.

#### 4.3.2 Data extraction and management

A data extraction form was developed based in part on MECCIR reporting standards (Methods Group of the Campbell Collaboration, 2019) and the PRISMA‐S Extension for reporting literature searches in systematic reviews (Rethlefsen et al., 2021), and includes bibliographic data (title, author, year), and information about the sources searched, the search strategies, and other methods related to search and retrieval. An initial draft of the form was piloted by six authors on nine different systematic reviews in a first stage of refinement. Based on this initial test, the form was modified to include a total of 79 items (Supporting Information Appendix 1). The form will be additionally piloted by at least two authors independently on a minimum of five systematic reviews. Further modifications to the data extraction form may be made based on this second pilot phase, and additional piloting will be conducted until a sufficient level of agreement between independent reviewers is reached.

Data about the following will be collected:

1. Bibliographic characteristics describing the review including title, authors, year of publication and the Campbell coordinating group associated with the review, as well as the date the search was conducted.
2. Sources searched including databases and platforms, a qualitative assessment of whether core bibliographic databases for the discipline were searched, the types of gray literature sources searched, and an assessment of whether geographic coverage was appropriate.
3. Supplementary searching methods including the use of free search engines, free scholarly databases (e.g., Google Scholar), hand‐searching and contacting experts.
4. The reproducibility of searches including reporting of details about the sources searched (e.g., database platform, date coverage, etc.), search strategy reporting, appropriate use of Boolean operators, text words and subject headings.
5. The use of reference management software and deduplication methods.
6. The involvement, or lack thereof, of an information specialist and the manner in which this is reported, and whether or not reference is made to the Campbell search methods guidance by Kugley et al. (2017).
7. The use of machine learning or automation tools for search and deduplication, as well as other emerging or less common methods.

Data will be extracted from all included Campbell systematic reviews using a form developed in Google Sheets. Data extraction will be carried out independently by two authors and discrepancies in the extracted data will be resolved by a third author. Where appropriate, data extractors will be provided a free text field in which to elaborate on multiple choice items to provide additional information and context.

#### 4.3.3 Data synthesis

Descriptive statistics will be used to understand the overall quality of search methods and reporting, and adherence to current standards. An assessment of search performance (e.g., precision vs. sensitivity, number of returned results) is beyond the scope of this study. A qualitative, narrative synthesis will describe any novel approaches identified and observations about trends in quality, biases, and reproducibility of the searches in Campbell systematic reviews. Searches conducted before and after the publication of Kugley et al. (2017) will be compared for methodological and reporting quality. Additional comparisons will be made between studies published by different Campbell Collaboration Coordinating Groups, and those reporting or not reporting the support of an information specialist. The percentage of items in the PRISMA‐S Extension Checklist reported will be recorded for each included study and will be used as a means for comparison.

#### 4.3.4 Summary of findings and assessment of the certainty of the evidence

##### Dissemination and use

Recommendations will be made based on the findings of the assessment to improve current standards for information retrieval, search peer review practices or guidance and support processes for Campbell authors. Common errors and deficiencies in reporting will be particularly noted and addressed through these recommendations. If new and emerging methods are found to be in practice, a need for guidelines on these methods will be highlighted. The findings and recommendations from this study will be circulated amongst Campbell Collaboration Coordinating Group editorial teams for feedback. Specifically, the editorial team of the Knowledge Translation Coordinating Group will be consulted to identify opportunities for addressing any shortcomings identified in the search methods and reporting of Campbell reviews through updates to guidance, improvements in the peer review process or communication and training of author teams. This assessment will be updated in a maximum of five years from the publication date to determine if any implemented recommendations have improved search methods and reporting.

## CONFLICT OF INTERESTS

Two authors (Sarah Young and Alison Bethel) are currently serving as co‐conveners of the Campbell Collaboration Information Retrieval Methods Group. Other authors (Sarah Young, Ciara Keenan, David Pickup, Zahra A. Premji) currently serve or have served as dedicated Campbell Collaboration coordinating group information specialists. Several authors have served as coauthors on Campbell Collaboration reviews. Those authors will not be involved in extracting data from systematic reviews in which they were involved.

## AUTHOR CONTRIBUTIONS

- Content: Sarah Young, Alison Bethel, Ciara Keenan, Kate Ghezzi‐Kopel, Elizabeth Moreton, David Pickup, Zahra A Premji, Morwenna Rogers, Bjørn Christian Arleth Viinholt.
- Systematic review methods: Sarah Young, Alison Bethel, Ciara Keenan, Kate Ghezzi‐Kopel, Elizabeth Moreton, David Pickup, Zahra A Premji, Morwenna Rogers, Bjørn Christian Arleth Viinholt.
- Statistical analysis: Not applicable.
- Information retrieval: Sarah Young, Alison Bethel, Ciara Keenan, Kate Ghezzi‐Kopel, Elizabeth Moreton, David Pickup, Zahra A Premji, Morwenna Rogers, Bjørn Christian Arleth Viinholt.

## PRELIMINARY TIMEFRAME

Approximate date for submission of the systematic review: January 1, 2021.

## PLANS FOR UPDATING THIS REVIEW

Considerations will be given to conducting an update of this methods assessment 5 years after the date of publication.

## Supporting information

Supporting information.

## References

[cl21208-bib-0001] Faggion, C. M. , Huivin, R. , Aranda, L. , Pandis, N. , & Alarcon, M. (2018). The search and selection for primary studies in systematic reviews published in dental journals indexed in MEDLINE was not fully reproducible. Journal of Clinical Epidemiology, 98, 53–61. 29476922 10.1016/j.jclinepi.2018.02.011

[cl21208-bib-0002] Franco, J. V. A. , Garrote, V. L. , Escobar Liquitay, C. M. , & Vietto, V. (2018). Identification of problems in search strategies in Cochrane reviews. Research Synthesis Methods, 9, 408–416. 10.1002/jrsm.1302 29761662

[cl21208-bib-0003] Koffel, J. B. , & Rethlefsen, M. L. (2016). Reproducibility of search strategies is poor in systematic reviews published in high‐impact pediatrics, cardiology and surgery journals: A cross‐sectional study. PLoS One, 11(9), e0163309. 27669416 10.1371/journal.pone.0163309 PMC5036875

[cl21208-bib-0004] Kugley, S. , Wade, A. , Thomas, J. , Mahood, Q. , Jørgensen, A.‐M. K. , Hammerstrøm, K. , & Sathe, N. (2017). Searching for studies: A guide to information retrieval for Campbell systematic reviews. Campbell Systematic Reviews, 13(1), 1–73.

[cl21208-bib-0005] Munn, Z. , Dias, M. , Tufanaru, C. , Porritt, K. , Stern, C. , Jordan, Z. , Aromataris, E. , & Pearson, A. (2021). The “quality” of JBI qualitative research synthesis: A methodological investigation into the adherence of meta‐aggregative systematic reviews to reporting standards and methodological guidance. JBI Evidence Synthesis, 19(5), 1119–1139. 10.11124/JBIES-20-00364 33989268

[cl21208-bib-0006] Page, M. J. , McKenzie, J. , Bossuyt, P. , Boutron, I. , Hoffmann, T. , Mulrow, C. D. , Shamseer, L. , Tetzlaff, J. , Akl, E. , Brennan, S. E. , Chou, R. , Glanville, J. , Grimshaw, J. , Hróbjartsson, A. , Lalu, M. M. , Li, T. , Loder, E. , Mayo‐Wilson, E. , McDonald, S. , … Moher, D. (2021). The PRISMA 2020 statement: An updated guideline for reporting systematic reviews. BMJ, 372, 371. 10.1136/bmj.n71 PMC8005924 33782057

[cl21208-bib-0007] Polanin, J. R. , Hennessy, E. A. , & Tsuji, S. (2020). Transparency and reproducibility of meta‐analyses in psychology: A meta‐review. Perspectives on Psychological Science, 15(4), 1026–1041. 10.1177/1745691620906416 32516081

[cl21208-bib-0008] Rethlefsen, M. L. , Kirtley, S. , Waffenschmidt, S. , Ayala, A. P. , Moher, D. , Page, M. J. , Koffel, J. B. , & PRISMA‐S Group (2021). PRISMA‐S: An extension to the PRISMA statement for reporting literature searches in systematic reviews. Systematic Reviews, 10(39), 1–9. 10.1186/s13643-020-01542-z 34285662 PMC8270366

[cl21208-bib-0009] The Methods Group of the Campbell Collaboration . (2019). *Methodological expectations of Campbell Collaboration intervention reviews: Reporting standards*. Campbell Policies and Guidelines Series.

[cl21208-bib-0010] Toews, L. C. (2017). Compliance of systematic reviews in veterinary journals with Preferred Reporting Items for Systematic Reviews and Meta‐Analysis (PRISMA) literature search reporting guidelines. Journal of the Medical Library Association, 105(3), 233–239. 28670210 10.5195/jmla.2017.246 PMC5490700

[cl21208-bib-0011] Wang, X. , Welch, V. , Li, M. , Yao, L. , Littell, J. , Li, H. , Yang, N. , Wang, J. , Shamseer, L. , Chen, Y. , Yang, K. , & Grimshaw, J. M. (2021). The methodological and reporting characteristics of Campbell reviews: A systematic review. Campbell Systematic Reviews, 17(1), e1134. 10.1002/cl2.1134 PMC8356304 37133262

[cl21208-bib-0012] Yoshii, A. , Plaut, D. A. , McGraw, K. A. , Anderson, M. J. , & Wellik, K. E. (2009). Analysis of the reporting of search strategies in Cochrane systematic reviews. Journal of the Medical Library Association, 97(1), 21–29. 10.3163/1536-5050.97.1.004 19158999 PMC2605027

