# Supplementary material for: PROTOCOL: Searching and reporting in Campbell Collaboration systematic reviews: An assessment of current methods
Source: Campbell Syst Rev. 2021 Dec 14;17(4):e1208. doi: 10.1002/cl2.1208 (PMC8988751; doi:10.1002/cl2.1208)
Supplement: Supplementary file 1 — Supporting information. [file CL2-17-e1208-s001.docx]

# Feedback

# Appendices

## 1 Data Extraction Form

##### Publication Information

1. Paper ID
2. Data Extractor (Name)
3. Title of Review
4. Authors
5. Year of Publication
6. Digital Object Identifier (DOI)
7. URL
8. Coordinating Group
9. Is this an update to a previously published review?
   1. Yes
   2. No
10. If Yes, were there changes to the search methods used? (If yes, extract. If no, stop here.)
11. Date of Search

##### Bibliographic Databases

11. Copy/paste text describing database searches (not including search strategy information)

12a. Were at least two databases relevant to the research question searched?

1. Yes
2. No

12b. If No, Please explain.

13. Notes/comments on database searches

##### Grey Literature

14. Copy/paste text describing grey literature searches (not including search strategy information)

15. Were conference proceedings searched?

1. Yes
2. No
3. Unclear

16. Were theses and dissertations searched?

1. Yes
2. No
3. Unclear

17. Were clinical trials searched?

1. Yes
2. No
3. Unclear

18. Was information from governments or inter-governmental organizations (e.g., WHO, UN, etc.) searched?

1. Yes
2. No
3. Unclear

19. Was information from non-governmental organizations searched (e.g., NGOs, donor agencies, think tanks, etc.)?

1. Yes
2. No
3. Unclear

20. Notes/Comments on grey literature searches

##### Geographic Coverage

21a. Is there an explicit argument made for the geographic coverage of the sources searched?

1. Yes
2. No

21b. If yes, copy and paste the text about geographic coverage of sources.

22a. Is the review topic limited to the US context only?

1. Yes
2. No

22b. If no, were databases searched that cover non-US-centric literature?

1. Yes
2. No
3. Unclear

22c. If no, were grey literature sources searched that cover non-US-centric information?

1. Yes
2. No
3. Unclear

##### Free Scholarly Databases

23. Google Scholar

1. Yes
2. No

24. Microsoft Academic

1. Yes
2. No

25. Dimensions

1. Yes
2. No

26. Other (insert name of database)?

27. Note/comments on searching of free scholarly databases

##### Search engines

28. Google

1. Yes
2. No

29. Other (insert name of search engine)?

30. Note/comments on use of search engines

##### Hand-searching

31. Was hand-searching conducted?

1. Yes
2. No
3. Unclear

32a. Were journal tables of contents hand-searched?

1. Yes
2. No
3. Unclear

32b. If yes, how many back issues or what date range of journals (or note if not specified)?

1. Yes
2. No
3. Unclear

33a. Were conference proceedings hand-searched?

1. Yes
2. No
3. Unclear

33b. If yes, how many back issues or what date range of proceedings (or note if not specified)?

##### Experts Contacted

34. Do the authors indicate that experts were contacted?

1. Yes
2. No

35. Do the authors indicate that practitioner networks such as listservs were contacted for additional studies?

1. Yes
2. No

##### Reporting of Database Searches

36. Do the authors report the date(s) the searches were run?

1. Yes
2. No

37a. Do the authors provide a complete list of all of the databases searched, including a list of all databases searched simultaneously on a single platform?

1. Yes
2. No

37b. If no, please explain.

38a. Do the authors report the platforms in which the databases were searched?

1. All
2. Some
3. None

38b. If not all, please explain.

39. Do the authors report the content date ranges for the databases searched?

1. All
2. Some
3. None

40. Did they provide a full line-by-line search strategy for at least one database (in the text, appendix or freely available external platform)?

1. Yes
2. No

41. Did they provide a full line-by-line search strategy for all databases (in the text, appendix or freely available external platform)?

1. Yes
2. No

42. Notes/comments on database search reporting

##### Quality of Database Searches

43a. For the reported searches, Boolean operators were used correctly.

1. Yes
2. No

43b. If Boolean operators were not used correctly, please explain.

44. Subject headings were used in databases with thesauri and controlled vocabulary

1. Yes
2. No

45a. Subject heading term explosion was used correctly

1. Yes
2. No

45b. If subject heading term explosion was not used correctly, please explain.

46a. Variations on keywords were searched (e.g., plural, alternate spellings)

1. Yes
2. No

46b. If variations on keywords were not searched, please explain.

47a. Multi-word phrases were searched using phrasing syntax (e.g., double quotation marks)

1. Yes
2. No

47b. If multi-word phrases were not searched using phrasing syntax, please explain.

48a. For the reported searches, specific database syntax was used correctly.

1. Yes
2. No

48b. If database syntax was not used correctly, please explain.

49. Date limits used

1. Yes
2. No

50.  Language limits used

1. Yes
2. No

51. Other limits/filters?

52a. Was the use of limits/filters justified?

1. Yes
2. No

52b. Please explain the justification or lack thereof of the use of limits/filters.

53a. Validated search filters were used.

1. Yes
2. No

53b. If validated search filters were used, copy and paste the relevant text here.

54a. Did the authors indicate that search strategies from other literature reviews were adapted or reused?

1. Yes
2. No

54b. If yes, was a citation provided?

1. Yes
2. No

##### Reporting of grey lit searches

55a. Do the authors provide a list of all grey literature sources searched?

1. Yes
2. No

55b. If no, please explain.

56. Grey literature searches reported

1. Exact search strategies reported in appendix only
2. General grey literature approach stated in text only
3. Exact searches in appendix and general approach described in text
4. No reporting of grey literature searching

##### Reporting of Free scholarly database searches

57. Details provided about how free scholarly database searches were performed

1. Exact search strategy/strategies reported
2. List of search terms provided but not exact strategy
3. Number of pages/results screened reported
4. 1 and 3
5. 2 and 3
6. No details given

58. Copy and paste the text describing free scholarly search engine searches

##### Reporting of search engines searches

59.  Details provided about how search engine searches were performed

1. Exact search strategy/strategies reported
2. List of search terms provided but not exact strategy
3. Number of pages/results screened reported
4. 1 and 3
5. 2 and 3
6. No details given

60.  Copy and paste the text describing search engine searches

##### Citation searching

61.  Was backward citation searching conducted?

1. Yes
2. No

62.  Were the references of related review articles searched?

1. Yes
2. No

63.  Was forward citation searching conducted?

1. Yes
2. No

64.  Was the method reported for forward citation searching?

1. Yes
2. No

##### Reference Management

65a.  Did the authors indicate that reference management software was used?

1. Yes
2. No

65b.  Copy and paste the text related to reference manager usage.

66. Do the authors describe the processes and software used to deduplicate records?

1. Yes
2. No

67. Do the authors document the total number of records retrieved from each database?

1. Yes
2. No

##### Involvement of Information Specialist

68.  Involvement of an information specialist

1. Lead author
2. Co-author
3. Mentioned in methods only (consulted but not acknowledged)
4. Mentioned in Acknowledgements only
5. Mentioned in methods and acknowledgements
6. No indication of information specialist involvement

69. Do the authors indicate that a peer review of the search strategy was performed?

1. Yes
2. No

##### Use of automation and machine learning tools

70.  Automation used for search term harvesting

1. Yes
2. No
3. Unclear

71.  Tool/Method used for search term harvesting

72.  Used for running of the searches

1. Yes
2. No
3. Unclear

73.  Tool/Method used for running searches

74.  Used for deduplication

1. Yes
2. No
3. Unclear

75.  Tool/Method used for deduplication

76.  Other uses?

### Other

77. Note any less common or emerging methods applied in any aspects of the search methods.

78.  Is Campbell Search Guidance document (Kugley et al 2017) referenced?

1. Yes
2. No

79. Was the search updated during the course of the review prior to publication?

1. Yes
2. No
